# Crosstalk between bone and muscle in chronic kidney disease

**DOI:** 10.3389/fendo.2023.1146868

**Published:** 2023-03-23

**Authors:** Limy Wong, Lawrence P. McMahon

**Affiliations:** ^1^ Department of Renal Medicine, Monash University Eastern Health Clinical School, Box Hill, VIC, Australia; ^2^ Department of Renal Medicine, Eastern Health, Box Hill, VIC, Australia

**Keywords:** osteokines, myokines, chronic kidney disease, crosstalk, bone, muscle

## Abstract

With increasing life expectancy, the related disorders of bone loss, metabolic dysregulation and sarcopenia have become major health threats to the elderly. Each of these conditions is prevalent in patients with chronic kidney disease (CKD), particularly in more advanced stages. Our current understanding of the bone-muscle interaction is beyond mechanical coupling, where bone and muscle have been identified as interrelated secretory organs, and regulation of both bone and muscle metabolism occurs through osteokines and myokines *via* autocrine, paracrine and endocrine systems. This review appraises the current knowledge regarding biochemical crosstalk between bone and muscle, and considers recent progress related to the role of osteokines and myokines in CKD, including modulatory effects of physical exercise and potential therapeutic targets to improve musculoskeletal health in CKD patients.

## Introduction

Chronic kidney disease (CKD) has a complex relationship with ageing, where the CKD phenotype provides an accelerated model of ageing through various mechanisms while ageing also hastens the progression of kidney disease. Patients affected by CKD experience profound musculoskeletal functional decline at a younger age, which is compounded by concurrent losses in bone and skeletal muscle mass, leading to reduced mobility and excessively high rates of falls and fractures, the effects of which are often life-limiting.

Disturbances in mineral and bone metabolism in CKD are conventionally jointly referred to as CKD-Mineral Bone Disorder (CKD-MBD), and comprises abnormalities in the homeostasis of calcium, phosphorus, vitamin D and parathyroid hormone (PTH); abnormalities of bone turnover, mineralisation or volume; and vascular or soft tissue calcification (1). Despite earlier research and vigorous exploration of therapeutic strategies in managing skeletal health focusing on bone and mineral abnormalities, a disturbing limitation in patient care persists, particularly in those with advanced CKD and/or who are dialysis-dependent. Meaningful clinical and biological targets are lacking in this population, resulting in management uncertainty for the prevention of bone loss and fractures.

Furthermore, it is increasingly recognised that sarcopenia plays a detrimental role in musculoskeletal health in CKD. Sarcopenia is a condition characterised by a reduction in muscle mass, strength and/or performance that accrues over many years and is associated with ageing (2). However, it is now recognised that sarcopenia begins earlier in life with many contributing factors beyond ageing alone. CKD patients suffer severe skeletal muscle wasting, and again this is particularly evident in its advanced stages. There is no pharmacological treatment available at present to reverse or halt the progression of muscle atrophy, although aerobic and resistance-training exercise and nutritional interventions have been shown to be of some benefit (3–5).

Our understanding of the interaction between bone and skeletal muscle now exceeds the concept of purely mechanical coupling, with evidence that these tissues communicate at a biomolecular level (6). Bone and muscle have been shown to be interrelated secretory organs, which produce osteokines (bone-derived factors) and myokines (muscle-derived factors) respectively. Each of these is important in regulating bone and muscle metabolism through autocrine, paracrine and endocrine signalling. The growing knowledge about the crosstalk between bone and muscle, and the likely influence of exercise on both tissues has important implications for clinical practice and introduces potential therapeutic targets to improve bone and muscle parameters, and consequently, the patient’s overall health and wellbeing. However, the biochemical relationship between bone and muscle in tandem is less well understood in the uraemic milieu.

This review presents an in-depth discussion about the known endocrine and other crosstalk between bone and muscle, the modulatory effects of physical exercise on these tissues, potential therapeutic targets and, lastly, important research questions in this field.

## Bone fragility in patients with chronic kidney disease

In the general population, osteoporosis is defined as a reduction in bone density; while bone disorders in CKD are more complex. The diagnosis and management of bone disorders in CKD is challenging for several reasons: (i) heterogeneous changes in bone tissue other than osteoporosis that could compromise bone strength such as osteomalacia, osteitis fibrosa cystica, adynamic bone disease (with inadequate bone turnover) and mixed bone lesions; (ii) the inability of dual-energy X-ray absorptiometry to provide meaningful details regarding underlying bone mineral density; (iii) inaccuracy of serum-derived bone turnover markers due to reduced renal clearance and (iv) infrequent use of bone biopsy, the gold standard determinant of bone pathology, due to its restricted availability, invasive nature, and limited interpretative expertise (7). Dialysis-dependent patients have a 4- to 14-fold higher risk of developing fractures than the healthy general population, a risk which extends to those with an estimated glomerular filtration rate (eGFR) between 15 to 60 mL/min/1.73m^2^ (8–12).

Despite early research efforts to identify better management strategies, the incidence of fractures has continued to rise in recent years, with no therapeutic agent yet approved for patients with kidney-related bone disease. Agents that have been targeted include vitamin D analogues and calcimimetics to suppress PTH and improve bone remodelling, thus potentially reducing fracture risk, and antiresorptive and osteoanabolic agents. The latter are approved for osteoporosis in the general population and have been administered off-label in CKD Stage 3B-5 high-risk patients. However, their use has been limited due to the lack of large-scale clinical trials and concern regarding their contribution to further kidney dysfunction and adynamic bone disease, which has now evolved as the predominant form of renal osteodystrophy associated with poor outcomes (13, 14).

## Muscle health in patients with chronic kidney disease

Skeletal muscle is the largest tissue in the human body, accounting for about 40-50% of body mass (15). It is imperative for gait and posture and also functions as an endocrine organ. Maximal muscle mass is achieved during young adulthood but after the age of 50, muscle loss occurs at a rate of ~1-2% per annum (16). Sarcopenia, a recently recognised disease entity, is common in older-aged adults but can also occur earlier in life from systemic illnesses, particularly conditions that trigger an inflammatory response such as CKD and malignancy. Sarcopenia is thus prevalent in the CKD population and is associated with an increased risk of hospitalisation and mortality in both dialysis and non-dialysis dependent patients. Low skeletal muscle mass (determined by radiological measures) is associated both with a higher waitlist mortality among kidney transplant candidates and an increase hospital readmission rate within the first 30 days after kidney transplant discharge (17, 18).

Several risk factors have been proposed to contribute to the development of sarcopenia in CKD including ageing, chronic inflammation, hormonal changes/resistance, metabolic acidosis, a more sedentary lifestyle and poor nutritional status leading to an imbalance between protein synthesis and degradation. We recently reported that the differentially expressed genes and proteins in skeletal muscle of CKD subjects belong to 8 major biological and signalling pathways, namely apoptosis, autophagy, inflammation, insulin/insulin-like growth factor 1 (IGF1) signalling, lipid metabolism, mitochondrial function, muscle cell growth and differentiation, and protein turnover (19).

## Bone metabolism and remodelling

The overall composition of bone tissue is altered in CKD due to abnormal systemic mineral metabolism and bone remodelling. Cells within bone include osteocytes (90-95%), osteoblasts (5%), and osteoclasts (1%) (20). It is a dynamic tissue and its structural integrity is maintained by bone remodelling, consisting of coordinated actions of the three cell types in a process tightly regulated by both local and systemic factors (21). The presence of the myogenic interleukin-6 (IL-6) activates the secretion of osteoblast and osteocyte-induced receptor activator of nuclear factor-κB (RANK), which drives osteoclastogenesis (22). This also results in an increased expression of RANKL, which binds to its receptor and triggers a cascade of signalling events that induce osteoclast differentiation, activation and survival. By contrast, osteoprotegerin (OPG), a soluble decoy receptor, binds RANKL to prevent the latter binding to RANK, thereby inhibiting osteoclastogenesis (23) and restraining bone loss. Dysregulation of the RANK-RANKL-OPG axis can lead to osteoporosis. Studies investigating RANKL levels in CKD patients have demonstrated conflicting findings (24–26), whereas OPG concentrations have consistently been reported to be higher in haemodialysis patients (24, 27, 28), which could reflect a compensatory protective mechanism to moderate bone remodelling.

## Osteokines and muscle metabolism

The discovery that osteocytes produced fibroblast growth factor 23 (FGF23), which circulates in different forms targeting the kidney and other organs including muscle, led to the recognition of bone as an endocrine organ. The list of osteokines has since continued to expand. Herein, we describe several osteokines that have been demonstrated to have regulatory effects on muscles (see **Table 1**).

**Table 1:** Biochemical crosstalk between bone and skeletal muscle.

Myokines	Effect on muscle	Effect on bone formation	Effect on bone resorption	Effect from exercise	Osteokines	Effect on bone formation	Effect on bone resorption	Effect on muscle	Effect from exercise
Myostatin	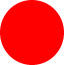	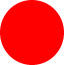	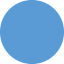	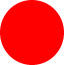	FGF23	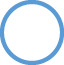	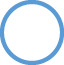	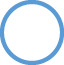	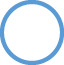
Irisin	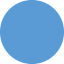	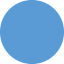	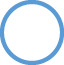	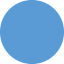	Osteocalcin	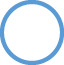	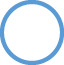	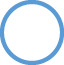	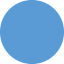
IL-6	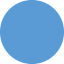	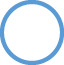	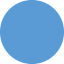	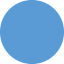	RANK/RANKL	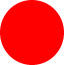	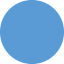	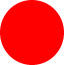	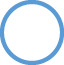
IL-15	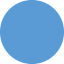	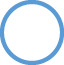	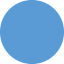	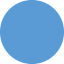	OPG	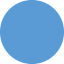	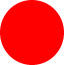	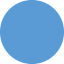	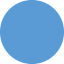
FGF2	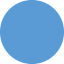	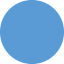	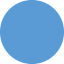	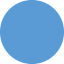	Sclerostin	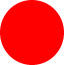	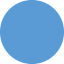	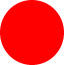	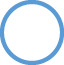
IGF-1	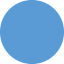	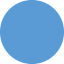	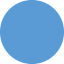	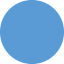	IGF-1	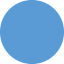	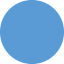	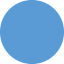	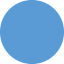

FGF2, fibroblast growth factor 2; FGF23, fibroblast growth factor 23; IGF-1, insulin-like growth factor-1; IL-6, interleukin-6; IL-15, interleukin-15; OPG, osteoprotegerin; RANK, receptor activator of nuclear factor-κB; RANKL, receptor activator of nuclear factor-κB ligand.

Red filled circles indicate inhibition/downregulation, blue filled circles indicate stimulation/upregulation and empty circles indicate uncertain effects or conflicting findings based on current literature.

### Fibroblast growth factor 23

FGF23 was first discovered in 2000 as a cause of autosomal dominant hypophosphataemic rickets (29). It is part of a superfamily of 22 peptides grouped into 7 subfamilies. FGF23 is mainly secreted by osteocytes and osteoblasts. It downregulates the luminal expression of sodium/phosphorus co-transporters in the proximal renal tubules to stimulate phosphaturia (30). FGF23 also suppresses the production of 1,25(OH)_2_Vitamin D by inhibiting 1-alpha hydroxylase, leading to phosphate wasting (31) and consequently poor bone mineralisation. The canonical FGF23 signalling pathway requires the obligatory co-receptor alpha klotho (α-KL), a transmembrane protein with extracellular glucuronidase activity, for binding to the first of four tissue-specific fibroblast growth factor receptors (FGFR), FGFR1 (32). However, some FGF23 signalling occurs independent of α-KL and is referred to as non-canonical FGF23 signalling, through binding and activation of other receptors: FGFR3/FGFR4/calcineurin/nuclear factor of activated T-cells (33), mainly in the setting of markedly elevated circulating FGF23 levels. Effects include distinct changes in several organs. Treatment of neonatal rat ventricular myocytes for 48 hours with varying concentrations of FGF23 induced morphometric hypertrophy in a similar extent to treatment with fibroblast growth factor 2 (FGF2). Moreover, *in vivo* experiments using both intravenous and intramyocardial injection of FGF23 showed induction of left ventricular hypertrophy in non-CKD mice (34).

What effects FGF23 has *in vivo*, with or without α-KL, remains uncertain. Higher serum levels have been shown to be independently associated with pre-frailty and frailty in a large cohort of community-dwelling elderly inhabitants (35). Similarly, as part of the SPRINT trial, Jovanovich et al. reported that FGF23 was associated with increased frailty among older adults with CKD (36), suggesting that FGF23 might have more diverse negative biological effects. Both FGF23 and α-KL were previously shown to inhibit differentiation of cultured skeletal muscle cells through downregulation of insulin/IGF-1 signalling (37). FGF23 was also found to induce premature senescence of human skeletal muscle mesenchymal stem cells *via* the p53/p21 pathway in an α-KL-independent manner, supporting its inhibitory effects (38). However, plasma FGF23 concentrations have also been reported to be positively correlated with muscle mass indices in a small haemodialysis cohort (39). As a potential therapeutic target, exercise endurance was found to improve significantly in C57BL/6J mice following exogenous administration of recombinant FGF23 (40). However, Avin et al. found that FGF23 did not influence C2C12 myoblast proliferation and differentiation and *ex-vivo* FGF23 treatment did not alter soleus contractility (41). Thus, the regulatory effect of FGF23 on skeletal muscle remains unresolved and further research is required to address this question.

### Osteocalcin

Osteocalcin (OCN) is the most abundant non-collagenous osteoblast-derived protein. There are two main forms of OCN in the circulation: γ−carboxylated and uncarboxylated (uOCN). Considered to be the active form, the latter has been shown to be involved in the regulation of insulin secretion and sensitivity (42), glucose metabolism (43), male fertility (44) and brain function (45). Although the data from *in vitro* and *in vivo* studies are controversial (46–49), OCN is thought to play an important role in bone remodelling by modulating osteoblast and osteoclast activity. High levels of circulating OCN and uOCN were observed in CKD patients (50–52), potentially due to increased bone metabolism, decreased renal clearance or both, with progression correlating with serum intact PTH and alkaline phosphatase (ALP) (50, 51), most probably reflecting the severity of the underlying bone disorder.

OCN was also discovered to promote muscle uptake and utilisation of glucose and fatty acids. *Ocn* ^-^/^-^ mice were found to have impaired exercise capacity, which was rescued by exogenous OCN administration (53). OCN was also shown to be a major regulator of IL-6 expression in the muscle during exercise and the rise in circulating IL-6 levels was proposed to originate from muscle, eventually forming a feed-forward axis to amplify adaptation to exercise (53). Moreover, using mice lacking OCN (*Ocn* ^-^/^-^), its receptor in all cells (*Gprc6a^-^/^-^*) and specifically in myofibres (*Gprc6a_Mck_* ^-^/^-^), Mera et al. showed that OCN signalling is essential in maintaining muscle mass by promoting protein synthesis in myotubes (54). uOCN was later found to enhance C2C12 myoblast cell proliferation and differentiation through activation of the PI3k/Akt, p38 MAPK and GPRC6A-ERK1/2 signalling pathways (55). Taken together, these findings strongly support that OCN, especially its active form, plays an important role in regulating muscle mass and might potentially be a therapeutic target in sarcopenia. However, recent studies have found to be contrary. Using a newly generated *Ocn*-deficient mouse model by deleting *Bglap* and *Bglap2*, Moriishi et al. showed that OCN played no role in bone formation or resorption, glucose metabolism, testosterone synthesis, or muscle mass (56). Similarly, Diegel et al. generated *Ocn*-deficient mouse using a CRISPR/Cas9-mediated gene editing tool and found that these mice displayed normal bone mass, serum glucose and fertility (57). The apparent discrepancies between studies remain inexplicable and additional efforts are required to confirm these findings.

### Receptor activator of nuclear factor-κB/Receptor activator of nuclear factor-κB ligand/Osteoprotegerin

RANK and RANKL are expressed in osteoclasts and osteoblasts respectively, as well as in skeletal muscle, and their interaction activates the nuclear factor-κB (NF-κB) signalling pathway, a key transcription factor inducing the expression of various proinflammatory genes, which can inhibit myogenic differentiation and activate the local ubiquitin proteasome system, ultimately leading to muscle atrophy (58). In addition, RANK has been shown to regulate calcium storage and muscle performance during denervation (59). Both genetic deletion of muscle RANK and short-term anti-RANKL treatment were shown to improve muscle integrity and strength of young dystrophic *mdx* mice (60). Hamoudi et al. demonstrated that anti-RANKL treatment inhibited NF-κB signalling and increased the proportion of M2 macrophages in dystrophic mice, thus reducing muscle inflammation and improving its mechanical properties (61). Similar findings were also observed in young dystrophic *mdx* mice when treated with recombinant full length OPG-Fc, a decoy receptor for RANKL (62). Furthermore, postmenopausal women who were treated with denosumab, a neutralising antibody against RANKL, for an average duration of 3 years were found to have improved appendicular lean mass and handgrip strength and these gains were absent in the bisphosphate treatment group (63). Altogether, it seems that the RANK-RANKL-OPG axis plays a pivotal role in bone and muscle metabolism. Given that coexistence of osteoporosis and sarcopenia is prevalent in the elderly population, the potential benefit of anti-RANKL treatment in possibly mitigating skeletal muscle atrophy while enhancing bone mechanical properties should be further investigated. However, any relationship between higher OPG concentrations (and OPG/RANKL ratio) and sarcopenia in CKD is yet to be determined.

### Sclerostin

Sclerostin is primarily secreted by mature osteocytes (64) and is a negative regulator of bone formation *via* inhibition of the Wnt/β-catenin pathway through binding to Wnt coreceptors, low-density lipoprotein receptor-related proteins 5 and 6 (65). Wnt-3a was also found to promote C2C12 myoblast differentiation through upregulation of *MyoD* and *Myogenin* while sclerostin treatment inhibited the effect of Wnt-3a on the C2C12 myoblast differentiation (65). A recent cross-sectional study of 240 healthy non-diabetic Korean individuals found that serum sclerostin levels were significantly higher in the low muscle mass group (66) and similar findings were observed in haemodialysis patients with diabetes (67). Interestingly, in a breast cancer mice model with bone metastasis, treatment with anti-sclerostin antibody prevented tumour growth in bone and bone destruction, as well as improvement in muscle function (68). Romosozumab, a human monoclonal antibody directed against sclerostin, has recently been approved for treatment of osteoporosis in postmenopausal women with high fracture risk. Its effect on skeletal muscle remains to be confirmed in larger human studies. Interestingly, skeletal muscle has also been found to secrete sclerostin, which works synergistically with bone-derived sclerostin to strengthen the negative regulatory mechanism of osteogenesis (69).

### Insulin-like growth factor 1

IGF1 is an anabolic hormone with about 50% structural homology with proinsulin. It is primarily synthesised in the liver, but also in extrahepatic tissues including bone and acts on skeletal muscle in a paracrine manner, primarily through the Type 1 IGF receptor (IGF1R) to stimulate cellular uptake of glucose and amino acids, enhance protein synthesis and suppress protein degradation (70). It is an important determinant of muscle mass and function. pAkt, which is a major cellular signalling effector of insulin and IGF-1, was consistently found to be reduced in the skeletal muscle of CKD individuals (19). A reduction of Akt activity induces activation of FOXO transcription factors, ultimately resulting in overexpression of genes that are involved in catabolic processes as well as autophagy (71). Moreover, reduced IGF1 concentrations in CKD patients have been associated with body composition and lower bone mineral density (72, 73). However, IGF1 therapy has not been shown to have beneficial effects on bone density, muscle strength or muscle mass in older women (74).

## Myokines and bone metabolism

Muscle-derived factors are called myokines, a term first proposed by Pedersen and colleagues in 2010 (75). These molecules include but are not limited to myostatin, irisin, IL-6, IL-8, IL-15, leukaemia inhibitory factor, brain-derived neurotrophic factor, IGF-1 and FGF2 (76).

### Myostatin

Myostatin was the first myokine identified in 1997 (77) and is primarily produced in skeletal muscle. It is a highly conserved member of the transforming growth factor-β superfamily and is one of the most potent negative regulators of skeletal myogenesis. It inhibits muscle cell growth and differentiation by interacting with the activin type II receptors (ActRIIA and ActRIIB), leading to upregulation of the cytokines and other signalling mediators that disrupt protein metabolism (71). Myostatin knockout results in excessive skeletal muscle hypertrophy in mice (78, 79) and notably, myostatin deficiency also increases bone mineral density (80–82), which might be attributed to both loading-associated effects and biochemical interaction between bone and muscle. Myostatin was later discovered to have a negative impact on bone remodelling by enhancing osteoclastogenesis and reducing bone formation (83). Enhancement of osteogenic differentiation was observed in bone-marrow derived mesenchymal stem cells from *Mstn-/-* mice as compared to wild-type mice, which was load-dependent (81). Furthermore, myostatin was shown to accelerate RANKL-mediated osteoclast formation *via* activation of the NFAT signalling pathway (84).

Several studies have investigated the plasma concentrations of myostatin in CKD patients, the majority of them reporting higher levels in CKD and dialysis-dependent patients compared to healthy subjects (85). A few novel myostatin-targeted agents such as LY2495655 (humanised myostatin antibody that neutralises myostatin) and bimagrumab (humanised monoclonal antibody that binds to ActRII) have been tested in Phase II clinical trials with inconsistent results; some demonstrating positive outcomes with increased lean body mass and improved handgrip strength and gait speed (86, 87), while others did not (88).

### Irisin

Irisin, a cleaved product of fibronectin Type III domain containing 5 (FNDC5), is secreted from skeletal muscle in response to an increased expression of peroxisome proliferator-activated receptor-γ co-activator-1-α (PGC1α) following exercise, to promote thermogenesis by browning white fat. It is also possibly involved in glucose metabolism (89). Irisin has been shown to promote myogenesis by enhancing myoblast proliferation and differentiation, increasing protein synthesis *via* activation of Akt and ERK, expanding the satellite cell pool and upregulating the expression of exercise-related genes, for example IL-6 (90, 91). Its anabolic effects on bone tissue are supported by *in vivo* studies, where low-dose weekly irisin injections for 4 weeks in young male mice resulted in increased cortical bone mass and strength, stimulating bone formation *via* upregulation of osteogenic transcription factors including activating transcription factor 4, Runt-related transcription factor 2 and Sp7 transcription factor. Interestingly, a lower number of osteoclasts were also observed in mice treated with irisin, which might contribute to the increase in bone strength (92). Recent findings raise the possibility that irisin could be a potential target for treating osteoporosis/CKD-MBD and sarcopenia. There is a known negative correlation between circulating irisin levels and osteoporotic fractures in postmenopausal women (93). Furthermore, plasma irisin levels are known to be lower in CKD patients (94), and recently, reduced irisin expression in the gastrocnemius muscle of 5/6 nephrectomised mice was found to be correlated with cortical bone mineral density (95).

### Interleukins

Some circulating inflammatory cytokines (e.g. IL-6, IL-7 and IL-15) are important for muscle development and growth as well as activation of muscle repair mechanisms. As mentioned previously, muscle-derived IL-6 promotes myogenesis and skeletal muscle growth *via* the regulation of proliferative capability of muscle stem cells (96). Apart from its glucose uptake and fatty acid oxidation, IL-6 stimulates bone resorption by inducing RANK and RANKL expression and the resorptive process has been demonstrated to be dependent on osteoblast signalling (22). *IL-6^-^/^-^* mice have increased bone formation and higher osteoclast numbers, but with a greater osteoclast apoptosis rate and reduction in resorption capacity (97). Its role in osteoblastogenesis remains controversial. Low grade chronic inflammation is prevalent in CKD patients and the interactions between cytokines, inflammation and muscle wasting are complex. On the one hand, systemic inflammation strongly correlates with muscle wasting, malnutrition, cardiovascular disease and mortality in patients with end-stage kidney disease (ESKD) (98–100). Higher expression of tumour necrosis factor-alpha (TNF-α) and IL-6 were observed in the muscle of CKD patients and mice compared to healthy controls and were associated with the development of muscle atrophy (19). Contrarily, both TNF-α and IL-6 have pleiotropic functions with a positive effect on muscle growth and regeneration. IL-6 was also shown to facilitate the local infiltration of macrophages and stimulate local IGF-1 production in muscle tissue of CKD mice (101). Furthermore, increased IL-6 efflux from muscle was found to correlate with increased muscle protein synthesis during haemodialysis (102). Similarly, IL-15, another anabolic factor in skeletal muscle, has also been demonstrated to have conflicting effects on osteoclast activity and bone mass (103, 104). Finally, higher circulating IL-15 levels correlate with a reduction in body fat and increased bone mineral content in mice (105).

### Others

Each of muscle-derived IGF1 and FGF2 exert anabolic effects on bone metabolism by promoting osteoblast proliferation and hastening bone formation. IGF1 regulates bone anabolism as a response to enhanced osteoblast survival and proliferation, whereas FGF2 has been proposed to be secreted following disruption of the plasma membrane in response to injury or mechanical muscle contraction, rather than by exocytosis (106). Moreover, FGF2 was found to reduce glucocorticoid-mediated bone resorption *via* inhibition of sclerostin signalling, reinforcing its anabolic effects on bone metabolism (107). Circulating FGF2 levels have been reported to be lower in patients with more advanced CKD (108, 109), though its role in the development of sarcopenia in CKD is yet to be determined.

## Effects of exercise on osteokines and myokines in chronic kidney disease

Observational studies have shown that patients with advanced CKD, particularly those on maintenance haemodialysis often have a sedentary lifestyle (110, 111) and about 45% of end-stage kidney disease (ESKD) patients report not performing any exercise at all (112). The health benefits of regular physical activity include a reduced risk of non-communicable diseases (e.g. heart disease, stroke and diabetes), better blood pressure control and improved mental health as well as overall quality of life. Physical activity improves physical function and reduces both pain and fall risk among adults with arthritis (113). In the CKD population, a highly active treatment group had a 25% risk reduction of all-cause mortality in comparison to inactive patients, even when factored for the presence of ESKD (114). Physical exercise also has a beneficial impact on bone mass, through promotion of bone formation and inhibition of bone resorption (115–117).

Several osteokines and myokines such as uOCN, OPG, irisin, IL-7, IL-15 and IL-6 are released in response to exercise training, exerting favourable physiological and metabolic effects in skeletal muscle and bone, in conjunction with a systemic anti-inflammatory effect (118, 119). In addition, both plasma and muscle myostatin levels were shown to decrease following aerobic and resistive training (120, 121). However, Gomes et al. investigated the effect of aerobic exercise on bone metabolism biomarkers (OCN, uOCN, sclerostin, PTH and total ALP) in non-dialysis CKD patients and found no differences in these biomarkers following a 24-week period of low-moderate intensity aerobic training except the total ALP (122). Similarly, both irisin and OCN levels were unaffected by resistance exercise in haemodialysis patients, though an increase in OPG was observed (123, 124). Yet, Zhou et al. reported higher plasma myostatin levels following 12 months of exercise training in a cohort of 151 non-dialysis-dependent CKD patients (125). These contrasting results underline the need for further studies in determining the effects of exercise (aerobic, resistance or alternative forms) on these bone-muscle biomarkers in CKD. Notwithstanding inconsistent findings from previous studies, a recent systematic review investigating the effects of physical activity in CKD patients reported beneficial effects of resistance exercise on bone health (126).

## Conclusions and directions

Over the last thirty years, studies in CKD patients have mostly focused either on bone or on muscle separately without recognition of an interplay between the two organs. This oversight has perhaps driven the bias and associated interpretative limitations of mechanical coupling. A better understanding of the secretory crosstalk and associated biochemical coupling between these two organs represents a subject of great interest, with conceivable potential in identifying novel therapeutic targets and ultimately addressing the major unmet needs in managing renal osteodystrophy and sarcopenia in CKD population.

That said, the well-documented beneficial effects of exercise on bone and skeletal muscle in CKD cannot be understated. Although many dialysis-dependent patients might be too frail to engage in vigorous exercise, a less intense regimen may still be valuable. It is of note that the incorporation of exercise programs into standard clinical care has been slow, possibly because of feasibility concerns (127). However, in line with the World Health Organisation 2018-2030 Global Action Plan to promote physical activity for each according to their ability across the life course, health professionals play an essential role in improving access and quality of health care in the CKD population. There are also potential opportunities for digital innovations to promote and support participation in physical activity to improve the health and well-being of our patients.

To finish, several key questions remain unanswered: (1) Is there a connection between osteoporosis, renal osteodystrophy and sarcopenia? (2) Does one condition precede or metabolically influence the other? (3) Is the phenotypic loss of muscle tissue simply related to comparable changes in bone over the course of CKD progression, each profoundly influenced by uraemia and a profoundly sedentary lifestyle? (4) Are other tissues, notably adipocytes, involved in the observed deleterious changes to bone and muscle in CKD?

## Author contributions

LW contributed to conception and design of the article. LW wrote the first draft of the manuscript. All authors contributed to the article and approved the submitted version.
